# Maxillofacial fractures among Sudanese children at Khartoum Dental Teaching Hospital

**DOI:** 10.1186/s13104-016-1934-5

**Published:** 2016-02-23

**Authors:** Hatim M. Almahdi, Mohammed A. Higzi

**Affiliations:** Faculty of Dentistry, University of Science and Technology, P.O. Box 30, Omdurman, Sudan; Faculty of Dentistry, University of Khartoum, P.O. Box 102, Khartoum, Sudan

## Abstract

**Background:**

Maxillofacial fractures in children are less frequent compared to adults but result in special complications affecting the growth, function and esthetics.

**Aim:**

The study aimed at assessing the characteristics and the pattern of facial fractures among children seen at Khartoum Teaching Dental Hospital (KTDH).

**Method:**

The study included 390 patients presenting with maxillofacial trauma at KTDH during a year period (2010–2011).

**Results:**

A total of 390 patients, diagnosed with facial fractures, were seen at KTDH; 14.1 % (55) were children below 16 years of age with the mean age of 10 years (SD ± 3.9). The ratio of males to females was 2.2:1. Most fractures were due to road traffic accidents (RTA) 56.4 %, followed by daily living activities 21.8 % and assault 16.4 %. The most prevalent anatomic sites of fractures were mandible 77 %; combination fractures i.e. more than one site 32.7 % and zygomatic-complex (13.5 %). Concomitant injuries were found in 9.1 %. Almost half of the patients were managed conservatively 49.1 %, closed reduction 34.5 % and surgical open reduction 16.4 %.

**Conclusions:**

The findings of this study indicated that pediatric facial fractures constitute 14.1 % of the total number of facial fractures. RTA was the main cause, which should be considered in legislative and preventive strategies.

## Background

Trauma is one of the leading causes of mortality and morbidity among children [1]. Trauma in children is usually accompanied with special complications such as the risk of growth disturbances, facial asymmetry e.g. temporomandibular joint ankylosis, and growing skull fracture [2]. Trauma-induced maxillofacial injuries in children may affect function as well as esthetic. They must be diagnosed promptly and managed appropriately to avoid disturbances of future growth and development.

The occurrence of maxillofacial fractures in those younger than 16 years of age ranges between 1 and 14.7 % of all facial fractures [1, 3, 4]. Worldwide the incidence of facial fractures is higher in boys than girls. The most frequent causes of facial fractures in children include daily life activities (e.g. playing, falls, sports related,), road traffic accident (RTA), and assaults [4–7].

The site and pattern of a fracture depends on the inter-relationship between etiology and force of the injury as well as features of the child’s stage of development. Mandibular fractures are the most common facial fractures while mid-face fractures are rare. In addition, children who sustain facial injuries in high-velocity vehicles in RTA are at increased risk for concomitant injuries (chest, abdomen, extremity and cervical spine) [1, 3].

Diagnosis and management of pediatric facial fractures is sometimes difficult compared to adults. It requires special attention to several critical factors related to anatomical, physiological and psychological development as well as the complications of trauma. Thus management techniques should be modified to address these special characteristics of pediatric facial fractures. This study aimed to describe the pattern and etiology of facial fractures in Sudanese children below 16 years.

## Methods

This is an observational, cross-sectional study that included pediatric patients with maxillofacial trauma who presented to Khartoum Teaching Dental Hospital (KTDH) during a year period (2010–2011).

All pediatric patients presenting to KDTH emergency unit with trauma were clinically examined and have undergone radiographic investigations including X-rays, and/or computed tomography scan (CT scan) as needed.

Patients that were diagnosed with maxillofacial fractures and were below 16 years of age were included in the study.

Demographic data was collected as well as the type of fracture, concomitant injuries, the possible causes of trauma, and treatment modality.

Mandibular fractures were classified according to Killey: condyle, ramus, angle, body, symphsyal and parasymphsyeal [8]. The mid-facial fractures sites were classified as: Le Fort (including all levels), zygomatic complex, naso-orbital-ethmoid (NOE) and others. Dentoalveolar fractures and other dental injuries were excluded.

The treatment of pediatric maxillofacial fractures was either conservative management (no intervention) or surgical management. The latter included closed reduction—with arch bars, eye loops, and maxillo-mandibular fixation (MMF)—or open reduction (rigid fixation with plates, miniplates, and screws).

Data analyzed by using statistical software package (SPSS version 15) for Windows (SPSS Inc., Chicago, IL, USA). Analysis was performed at accepted level (*p* value) of 0.05. Descriptive analyses: proportions, percentages, and frequency distribution were performed.

All study protocols were approved by the ethical committee of the University of Khartoum, Faculty of Dentistry. Informed consents were obtained from the guardians of all patients included in the study.

## Results

During the study period (12 months), a total of 390 patients with facial fractures were seen at KTDH; 14.1 % (55) of them were children below 16 years of age. The mean age is 10 ± 3.9 years and range (3–16 years). Most facial fractures 47 % were in the age group 12–16 years, followed by 29.1 % in the age group 7–11 years, while the least number of fractures were seen in 23.6 % in the age group below 6 years. The male to female ratio among pediatric facial fractures was 2.2:1.

Most maxillofacial fractures among children were caused by RTA 56.4 %, followed by daily activities 21.8 %, assault 16.4 %, and others 5.5 %. Pediatric facial fractures caused by RTA were consistently increasing with age. There was a significant relationship between the age and etiology of facial fracture (P = 0.003). Table 1. Males sustained 77.7 % of facial fractures which were due to assault Table 2.

**Table 1:** Frequency of etiology of facial fractures in relation to age to group

Age group (years)	RTA	DLA	Assault	Others	Total
Etiology of facial fractures					
0–6	7	5	0	1	13
7–11	11	4	0	1	16
12–16	13	3	9	1	26
Total	31	12	9	3	55

*RTA* road traffic accident, *DLA* daily living activity

**Table 2:** Distribution of frequency and percentages of etiology and gender

Etiology	Male	Female	Total
RTA	36.4 % (20)	20 % (11)	56.4 % (31)
DLA	14.5 % (8)	7.3 % (4)	21.8 % (12)
Assault	12.7 % (7)	3.6 % (2)	16.3 % (9)
Others	5.5 % (3)	(0)	5.5 % (3)

*RTA* road traffic accident, *DLA* daily living activity

Seventy four pediatric facial fractures were sustained in 55 patients. These were due to combination fractures (more than one fracture in one patient) and occurred in 32.7 % patients. Most of the facial fractures occurred in the mandible 76.8 %, followed by mid-face fractures; zygomatic complex fractures 13.5 %, Le Forte 6.7 % and NOE 1.3 %.

The concomitant injuries were seen in 9.1 % of children with facial fractures. These included skull, femur, humerus fractures and chest injuries. Forty percent were in the age group 7–11 years, and 60 % in the age group 12–16 years. All of them were males.

Intervention was in the form of conservative management in 49.1 % of the patients, 34.5 % closed reduction (e.g. simple wire, lingual splints) and 16.4 % open reduction (open reduction and internal fixation)

## Discussion

KTDH is the main trauma centre in Khartoum state (the capital of Sudan) which has about 5 million inhabitants; representing about 13 % of population of the country [9].

The pattern of maxillofacial fractures in children differs from adults because of the unique features of the pediatric patients’ jaws. The child’s face is proportioned quite different from that of the mature adult [11]. Maxillofacial fractures presentation among pediatric patients were consistently influenced by geographic area, socioeconomic status of the population and the period of investigation [10–12].

In this study 14.1 % of the patients presenting with facial fractures were children. This figure is consistent with other studies from different parts of the world, where facial fractures in the pediatric population are reported to be less than 15 % of all facial fractures worldwide [4, 10, 11].

This study confirms that male children are prone to facial fractures more than females. This also agrees with other studies which reported higher occurrence of pediatric facial fractures in males than in females at all age groups. This male preponderance has been attributed to cultural and social restrictions for female outdoor activities without supervision in comparison to greater outdoor activities, and more dangerous physical activities and contact sports in the case of males. Another possible explanation for this could be that females mature earlier than males [3, 4, 13–15].

RTA was the most common etiological factor of facial fractures in this study as shown in Table 3. This is explained by the fact that Khartoum city is facing heavy traffic problems as there is an increasing number of cars (a car for each 120 citizens) coupled with badly maintained roads. This is further complicated by the mechanical condition of the cars, and few pedestrian bridges and crossings. The most recent report has estimated that there were around 44442 RTA in Khartoum state in the year 2009 [18]. This rise of RTA was explained by Kaban who described how RTA has increased in recent years, although falls and assaults remain steady [11, 16, 17]

**Table 3:** Distribution of frequency and percentages of site of mandibular fractures

Site	Percentage of fractures
Symphysis	19.3 % (11)
Parasymphysis	8.8 % (5)
Body	28.1 % (16)
Angle	14 % (8)
Condyle	29.8 % (17)
Total	100 % (57)

Daily living activity (playing, falls) was the second most common etiological factor. It occurred mostly in children below the age of 6 years. There was one patient in whom the fractures were caused by an animal (donkey). Another two cases were due to falling into a well while they were collecting water. These cases were peculiar to rural areas where children participate in the community activities and work. These are consistent with other studies that reported young children are prone to sustain injuries from low-velocity forces (e.g. falls), while older children are more likely to be exposed to high-velocity forces (e.g. RTA) [5, 17, 19].

Assaults and interpersonal violence as causative factors of facial fractures were seen in 16.4 % of the children. These are uncommon causes of facial fractures in children, but when they occur they are commonly seen in the older age groups as confirmed in this study. In addition, they mostly occur among males. This indicates an increasing frequency of assault-related accidents in this age group in particular. A significant increase in assault-related cases was observed in a series of study by Lida et al. in Japan and Thorén et al. in Finland where they showed that causes of facial fractures in children in the early teens gradually assume a pattern similar to that of adults [3, 7, 11].

Fractures of the mandibular condyle, either in isolation or in combination with other sites, were the most common mandibular fractures seen. In this study they occurred most commonly in the age group 7–11 years and decreased in older groups, which is consistent with other reports [3, 20]. This is attributed to the peculiar pediatric anatomic features such as the highly vascularised condyle and thin neck which are poorly resistant to low velocity trauma during falls [21]. Such fractures were important not only because of the high incidence of these injuries, but also because of the possible long-term adverse effects since they can affect facial growth and lead to ankylosis of temporomandibular joint (TMJ), and may also lead to the formation of a bifid condyle [22, 23].

In this study the mandibular body and angle fracture consistently increased with age and occurred exclusively in males as in other studies [20]. Lida et al. explained these types of fractures as being one of the common fracture sites in adults and this may reflect the changes of etiology, such as an increase of assault, as well as the growth of the mandible in this age group [17].

Most of the mid-face fractures reported were in the older age groups and due to RTA or assault. Thoren et al. mentioned that fractures in the mid-face were frequently multiple and/or comminuted. Furthermore, they cannot be classified to any particular Le Fort classification [7]. The low incidence of mid-face fractures is due to the protruding anatomic position of the mandible and the cranium. This provides protection and absorbs most of the traumatic impact in addition to the fact that the mid-facial bones are more elastic [1, 24]. The proportion of pediatric patients identified with midfacial fractures increased in recent years, probably due to the increased use of adequate imaging. Van As et al. concluded that conventional radiographs were not exact for diagnosis of mid-facial fracture [7, 12].

The management of pediatric fractures at KDTH in this study reflects the tendency towards non-surgical procedures whenever possible except in severe multiple or comminuted fractures where surgical intervention is mandatory. That may be attributed to economic reasons where the cost leads patients and surgeons to choose less expensive treatment modalities. It also reflects the hot debate about surgical management of fractures in growing bones and its effect. Some authors advocates for conservative treatment of the growing bones whenever possible [20]. Others argue for the usefulness of open reduction and plate fixation in children and encourages removal of titanium plates after fracture healing [25]. Iatrou et al. stated that surgical treatment of fractures in adults has been established and documented, although more conservative treatment is still used in countries with poor resources. Closed reduction was selectively applied in condyle fractures [25].

## Conclusions

The findings of this study have shown that the pediatric facial fractures in KTDH constitute 14.1 % of the total facial fractures. RTA and assault are the rising cause of pediatric fractures. Mandibular fractures are predominating, and in particular condylar fractures with all its morbidity.

### Bullet Points

- The prevalence of the pediatric facial fractures is substantial and needs special consideration in this special subpopulation.
- More research and training is needed on the different treatment modalities considering the serious consequences of trauma and treatment to growing children.
- RTA is the main cause; hence more effort in legislative and preventive areas is needed.

## Abbreviations

KTDH: Khartoum Dental Teaching Hospital
RTA: road traffic accident
MMF: maxillo-mandibular fixation
